# The clinical significance of adenomatous polyposis coli (APC) and catenin Beta 1 (CTNNB1) genetic aberrations in patients with melanoma

**DOI:** 10.1186/s12885-021-08908-z

**Published:** 2022-01-05

**Authors:** Georgia Sofia Karachaliou, Rached Alkallas, Sarah B. Carroll, Chongshan Caressi, Danny Zakria, Nirali M. Patel, Dimitri G. Trembath, Jennifer A. Ezzell, Guillaume J. Pegna, Paul B. Googe, Jonathan P. Galeotti, Fatih Ayvali, Frances A. Collichio, Carrie B. Lee, David W. Ollila, Margaret L. Gulley, Douglas B. Johnson, Kevin B. Kim, Ian R. Watson, Stergios J. Moschos

**Affiliations:** 1 Department of Medicine, The University of North Carolina at Chapel Chapel Hill, Chapel Hill, NC USA; 2 Department of Biochemistry, McGill University, Montreal, QC, Canada; 3 California Pacific Medical Center Research Institute, San Francisco, CA USA; 4 Department of Medicine, Vanderbilt-Ingram Cancer Center, Nashville, TN USA; 5 Department of Pathology & Laboratory Medicine, The University of North Carolina at Chapel Chapel Hill, Chapel Hill, NC USA; 6 Lineberger Comprehensive Cancer Center, The University of North Carolina at Chapel Hill, Chapel Hill, NC USA; 7 Department of Cell Biology & Physiology, Histology Research Core Facility, The University of North Carolina at Chapel Hill, Chapel Hill, NC USA; 8 Department of Dermatology, The University of North Carolina at Chapel Chapel Hill, Chapel Hill, NC USA; 9 Department of Surgery, The University of North Carolina at Chapel Chapel Hill, Chapel Hill, NC USA

**Keywords:** Melanoma, Central nervous system neoplasms, Genetic markers, Immunomodulation, Immunotherapy

## Abstract

**Background:**

Melanoma-intrinsic activated β-catenin pathway, the product of the *catenin beta 1* (*CTNNB1)* gene, has been associated with low/absent tumor-infiltrating lymphocytes, accelerated tumor growth, metastases development, and resistance to anti-PD-L1/anti-CTLA-4 agents in mouse melanoma models. Little is known about the association between the *adenomatous polyposis coli (APC)* and *CTNNB1* gene mutations in stage IV melanoma with immunotherapy response and overall survival (OS).

**Methods:**

We examined the prognostic significance of somatic *APC*/*CTNNB1* mutations in the Cancer Genome Atlas Project for Skin Cutaneous Melanoma (TCGA-SKCM) database. We assessed *APC*/*CTNNB1* mutations as predictors of response to immunotherapies in a clinicopathologically annotated metastatic patient cohort from three US melanoma centers.

**Results:**

In the TCGA-SKCM patient cohort (*n* = 434) presence of a somatic *APC*/*CTNNB1* mutation was associated with a worse outcome only in stage IV melanoma (*n* = 82, median OS of *APC*/*CTNNB1* mutants vs. wild-type was 8.15 vs. 22.8 months; log-rank hazard ratio 4.20, *p* = 0.011). *APC*/*CTNNB1* mutation did not significantly affect lymphocyte distribution and density. In the 3-melanoma institution cohort, tumor tissues underwent targeted panel sequencing using two standards of care assays. We identified 55 patients with stage IV melanoma and *APC*/*CTNNB1* genetic aberrations (mut) and 169 patients without (wt). At a median follow-up of more than 25 months for both groups, mut compared with wt patients had slightly more frequent (44% vs. 39%) and earlier (66% vs. 45% within six months from original diagnosis of stage IV melanoma) development of brain metastases. Nevertheless, time-to-development of brain metastases was not significantly different between the two groups. Fortunately, mut patients had similar clinical benefits from PD-1 inhibitor-based treatments compared to wt patients (median OS 26.1 months vs. 29.9 months, respectively, log-rank *p* = 0.23). Less frequent mutations in the *NF1*, *RAC1*, and *PTEN* genes were seen in the mut compared with wt patients from the 3-melanoma institution cohort. Analysis of brain melanoma tumor tissues from a separate craniotomy patient cohort (*n* = 55) showed that melanoma-specific, activated β-catenin (i.e., nuclear localization) was infrequent (*n* = 3, 6%) and not prognostic in established brain metastases.

**Conclusions:**

*APC*/*CTNNB1* mutations are associated with a worse outcome in stage IV melanoma and early brain metastases independent of tumor-infiltrating lymphocyte density. However, PD1 inhibitor-based treatments provide comparable benefits to both mut and wt patients with stage IV melanoma.

**Supplementary Information:**

The online version contains supplementary material available at 10.1186/s12885-021-08908-z.

## Introduction

Despite identifying effective systemic treatments in metastatic melanoma (MM) [1, 2], post hoc subgroup analyses performed in several randomized clinical trials suggest that distinct, genetically defined patient subgroups may experience differential benefit from these therapies [3, 4]. Genetic aberrations in the *PTEN*, *RAC1*, *NRAS* and *EZH2* genes may affect overall survival (OS) via host-immune response regulation [5–8]. The Wnt/β-catenin pathway is known to regulate the immune response in colorectal and perhaps other cancer types (**Supplementary Fig.** **1** and [9]). Activation of β-catenin can lead to metastases in melanoma mouse models in cooperation with *BRAFV600E* mutation and *PTEN* inactivation [10]. In mouse melanoma models, activation of the β-catenin pathway in melanoma cells is associated with low/absent tumor-infiltrating lymphocytes (TILs) in tumors and resistance to anti-PD-L1/anti-CTLA-4 antibody therapy [11]. Among tumors that lack T-cell infiltration, activating somatic mutations in the *CTNNB1* gene that encodes β-catenin as well as somatic mutations in the gene encoding for the adenomatous polyposis coli gene (*APC*), a negative regulator of the β-catenin signaling pathway, accounts for approximately 75% of all genetic aberrations in the β-catenin signaling pathway [9]. Melanoma cell-derived or paracrine-derived wingless-type MMTV integration site 5a (Wnt5a), a WNT protein involved in Wnt signaling, can affect activation of the β-catenin pathway within nearby dendritic cells in a paracrine fashion and drive immune tolerance (**Supplementary File** **1****, Fig. S1** and [12]).

Despite solid preclinical evidence about the immunomodulatory role of the Wnt/β-catenin pathway, little is known about the association of genetic aberrations in the *APC* and *CTNNB1* genes with response to immunotherapies and prognosis in patients with MM [13]. In this study, we investigated the clinical significance, prognostic and predictive, of *APC* and *CTNNB1* genetic aberrations in melanoma patients. We present data from two independent melanoma patient cohorts in which the β-catenin pathway has been investigated by DNA sequencing of the *APC* and *CTNNB1* gene. Our results suggest that patients with MM bearing *APC*/*CTNNB1* genetic aberrations have a worse prognosis than patients without. However, analysis of a separate, clinicopathologically annotated, multi-institutional cohort of patients with MM suggests that patients with *APC*/*CTNNB1* genetic aberrations have a similar benefit from immunotherapies compared to patients without. Unexpectedly, patients with MM and *APC*/*CTNNB1* genetic aberrations demonstrate a slightly higher frequency and early (i.e., within the first six months) development of melanoma brain metastases (MBMs) compared to patients without. However, in a separate third cohort in which tumor tissues from patients who underwent craniotomy for MBMs were immunohistochemically stained with β-catenin, neither expression nor nuclear localization of β-catenin in melanoma cells have any prognostic significance. Similar to other, more frequent hotspot mutations in MM (*BRAFV600* and *NRASQ61*) [14–16], expression of these low-frequency *APC*/*CTNNB1* mutations may have an adverse prognosis, in part due to the development of brain metastases, but does not mitigate the clinical benefit from immunotherapies.

## Patients and methods

### The DNA sequencing patient cohorts

#### Patients and tumor specimens

We analyzed the following two patient cohorts whose tumor DNA had been sequenced for the presence of *APC* and *CTNNB1* genetic aberrations. The first cohort included patients with stage II, III, and IV melanoma from the Cancer Genome Atlas Database in Cutaneous Melanoma (TCGA-SKCM) [17]. The second cohort included patients with MM whose archived tumor specimen expressed genetic aberrations in the *APC* and/or *CTNNB1* genes using a DNA sequencing strategy (MM multi-institutional cohort). This latter cohort included patients from the Melanoma clinics in the University of North Carolina Hospitals at Chapel Hill (UNC-CH), Vanderbilt University, and the California Pacific Medical Research Institute (CPMRI, San Francisco, CA). In this cohort, we defined MM as the presentation of a known primary melanoma to non-regional lymph nodes, soft tissue (excluding satellite or in-transit disease; i.e., M1a), lung (M1b), visceral sites (M1c), or brain (M1d). Melanoma presentation to lymph nodes and soft tissue from an unknown primary were also considered MM. All methods were performed in accordance with the Declaration of Helsinki, were approved by the institutional review board (IRB) for each of UNC-CH (the University of North Carolina at Chapel Hill, 16–2959), Vanderbilt University (Vanderbilt University Medical Center, MEL 09109-Storage and Research Use of Human Biospecimens from Melanoma Patients), and the CPMRI (Sutter Health IRB), and waived the need for informed consent [18–20].

Regarding the TCGA SKCM cohort, we retrieved the following clinical data fields from the National Cancer Institute Genomic Data Commons Data Portal (https://gdc-portal.nc.nih.gov): *curated_TCGA_age_at_sample_procurement*, *sex*, *tumor_tissue_site*, *breslow_depth_value*, *melanoma_ulceration_indicator*, *malignant_neoplasm_mitotic_count_rate*, *Lymphocyte.density*, *curated_pathologic_stage*, *curated_days_to_last_followup*, and *curated_vital_status* [17]. We did not use the serum lactate dehydrogenase (LDH) data from the TCGA SKCM database for any downstream subgroup analysis of stage IV melanoma patients because serum LDH, a prognostic factor only for stage IV melanoma [21], was only available at diagnosis and not at specimen procurement. Using the *curated_pathologic_stage* and *tumor_tissue_site* data fields, we generated a new “clinical” stage that reflects the American Joint Committee on Cancer (AJCC) stage of patients at specimen procurement because we believe that this clinical AJCC stage at specimen procurement is a more reliable predictor of prognosis. For example, if for a given tumor, the *tumor_tissue_site* had been classified as *primary* and the *curated_pathologic_stage* had been classified as *stage IV* in the TCGA database, then the “clinical” AJCC of the patient’s melanoma at the time of the primary melanoma specimen procurement is stage IV (MM). Similarly, if the *tumor_tissue_site* had been classified as a *regional lymph node* and the *curated_pathologic_stage* had been classified as *stage IV* in the TCGA database, then the patient’s melanoma AJCC clinical staging was stage IV. If the *curated_pathologic_stage* was not available (NA) and the *tumor_tissue_site* had been classified as distant metastases, then the AJCC of the patient’s melanoma at the time of specimen procurement is stage IV.

We collected the following patient data from the combined UNC-CH/Vanderbilt/CPMRI cohort under the relevant IRB-approved guidelines and regulations: patient demographics (age, sex), melanoma subtype, clinicopathologic characteristics at original diagnosis, time from initial diagnosis of MM to initial diagnosis of brain metastases, OS from initial diagnosis of MM to the last follow-up, and survival status at last follow-up. Lastly, we collected data regarding clinical benefits from systemic immunotherapies and other non-immunotherapy treatments. The antitumor response was assessed in patients with any size of measurable lesions by computerized tomography, magnetic resonance imaging, or positron emission tomography scans. Subcentimeter tumor lesions were also considered measurable. Antitumor response was defined as shrinkage of measurable lesions to any degree without developing new lesions and growth of pre-existing ones, as long as responses were durable (> 6 months). If systemic treatment was administered as an adjuvant for no evidence of disease stage IV melanoma, the patient was considered a responder. We defined progression as either developing new lesions in stage IV or growth of pre-existing ones to any degree. Mixed responses (i.e., shrinkage of several lesions but growth of others) and non-durable responses (i.e., early responses followed by later progression) were considered progression. If a systemic treatment was administered as adjuvant therapy for no evidence of stage III melanoma and patient developed stage IV melanoma afterward, the patient was considered a progressor. Finally, a patient who may have progressed on a single-agent PD1 inhibitor but may have responded to ipilimumab-based treatment(s) was regarded as an overall responder.

#### Variant calling

We recently described variant calling for the complete TCGA-SKCM cohort [22]. Somatic mutation calls are available at the Github repository hosting service (https://github.com/ianwatsonlab/multiomic_melanoma_study_2019). The TruSight Tumor 26-gene Illumina Assay (UNC-CH patients only) includes probes covering the second transcribed exon (exon 3) of the *CTNNB1* gene and exon 15 of the *APC* gene [23, 24]. Details about variant calling as part of the FoundationOne CDx assay (UNC-CH, Vanderbilt University, and CPMRI) have been described elsewhere [25].

### The craniotomy patient cohort

#### Patients and tumor specimens

Under the UNC-CH IRB-approved protocol 16–2959, we analyzed tumor specimens corresponding to patients who underwent craniotomy for melanoma brain metastases (MBM) at UNC-CH. We have recently reported information about patient demographics, histopathologic data, and OS defined from craniotomy to the last follow-up, and status at last follow-up (alive or deceased) [26].

#### β-Catenin staining for single-color immunohistochemistry

We performed single-color immunohistochemistry (IHC) for β-catenin in 5 μm-thick sections obtained from formalin-fixed, paraffin-embedded melanoma craniotomy tissues placed on positively charged glass slides, as we have previously described [26]. Briefly, slides were dried, then baked at 60 °C for 90 min, followed by heat-induced epitope retrieval using HIER Buffer L (Thermo Scientific, TA-135-HBL, Thermo Fisher Scientific, MA). Endogenous peroxidases were blocked using 3% hydrogen peroxide for 10 min at room temperature (RT). Tissues were then blocked using 10% normal goat serum for 1 h at RT and incubated with an antibody against β-catenin (rabbit monoclonal, clone 247, ab32572, 1:500 dilution, Abcam, MA) overnight at 4 °C. For negative control, we stained representative tissue sections omitting the primary antibody. Following incubation with biotinylated goat anti-Rabbit IgG (111–065-144, dilution 1:500, Jackson ImmunoResearch Laboratories, PA) for 60 min at RT, tissues were treated with ABC-HRP (Vector, PK-6100, Vector Laboratories, CA) and visualized using ImmPACT VIP Peroxidase Substrate (Vector, SK-4605). Finally, tissues were counterstained with 0.5% Methyl Green, dehydrated, cleared, and cover-slipped using DPX (Electron Microscopy Sciences, 13,512, Electron Microscopy Science, PA).

### Statistical analysis

We used descriptive statistics to present important clinical and molecular characteristics of patients with *APC* and *CTNNB1* genetic aberrations in the two DNA sequencing patient cohorts (TCGA SKCM and the UNC-CH/Vanderbilt/CPMRI cohorts). We used Oncoprinter (www.cbioportal.org/ocoprinter) to visualize genomic data for both patient cohorts. In addition, we performed OS analysis using the Kaplan-Meier method to assess the prognostic significance of *APC*/*CTNNB1* genetic aberrations in the TCGA SKCM cohort for each AJCC clinical stage and in patients from the UNC-CH/Vanderbilt/CPMRI cohort who were treated with immunotherapies for stage IV melanoma. We performed OS analysis using Prism 8 (GraphPad Software, version 8.3.1, San Diego CA).

Given the favorable prognostic significance of the tumor mutation burden in MM [27], a Cox proportional hazard (coxph) regression model was used to study the prognostic value of *APC*/*CTNNB1* mutations in the TCGA SKCM patient dataset. This model was implemented in the “survival” package in R (r-project.org) and was fitted to right-censored survival intervals relative to specimen procurement time. In this model, we used the *APC*/*CTNNB1* somatic mutation status as a predictor variable (missense, in-frame indels, loss-of-function mutations), while controlling for tumor mutation burden (total number single nucleotide variants per sample, both untransformed and on log scale). Firth logistic regression implemented in the “logistf” R package, fitted using penalized maximum likelihood, was used to evaluate the co-occurrence of *APC*/*CTNNB1* somatic mutations with mutations in other melanoma driver genes. One model was fitted per melanoma driver gene, using the mutation status of the driver as the predictor and the mutation status of *APC*/*CTNNB1* genes as the response. Two models were fitted; one model including and the other model omitting tumor mutation burden as a covariate (log10-transformed). We computed the false discovery rate of the driver gene coefficient *p*-value independently for each set of models using the Benjamini-Hochberg method. We only considered likely impactful mutations when evaluating the mutation status of driver genes (missense, in-frame indels, loss-of-function mutations), but we included all single nucleotide variants when computing tumor mutation burden.

We analyzed the expression of β-catenin across different cellular compartments within the brain (i.e., melanoma, reactive glia, TILs, normal brain parenchyma) using the Wilcoxon matched-pairs signed-rank test, as we have previously described [26]. We investigated the correlation between protein expression of various proteins by melanoma cells and TIL density using the Kendall rank correlation statistic. We dichotomized protein expression of β-catenin in melanoma cells by IHC as high expression (2+, 3+) or low/absent expression (0, 1+) by IHC. We then performed an OS analysis to assess the prognostic significance of β-catenin in MBM using the Kaplan-Meier method, as we have previously described [26]. We performed statistical analysis using Prism 8 (GraphPad Software, version 8.3.1, San Diego CA).

## Results

### Prognostic significance of *APC*/*CTNNB1 somatic* mutations in cutaneous melanoma; the Cancer genome atlas cutaneous melanoma cohort

**Fig. 1:**
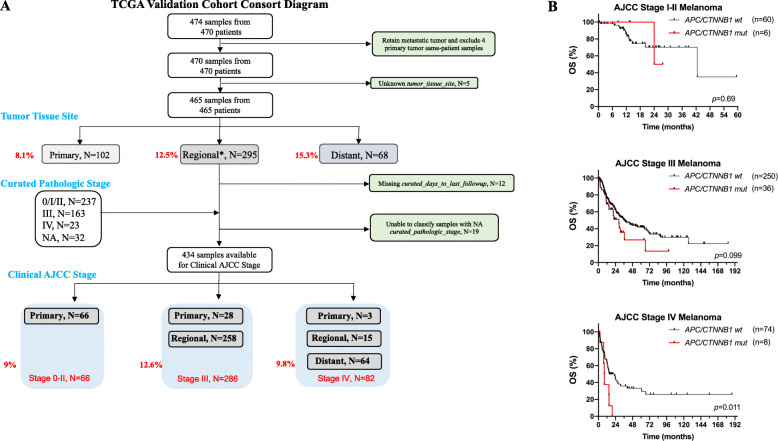
TCGA SKCM tumor specimens and *APC*/*CTNNB1* somatic mutation status. (**A**) CONSORT diagram of the 470 TCGA SKCM tumor specimens in relation to the *APC/CTNNB1* somatic mutation status, tumor tissue site, and AJCC stage at specimen procurement. (**B**) Overall survival analysis (Kaplan-Meier method) according to *APC/CTNNB1* somatic mutations and AJCC stage at specimen procurement

To investigate whether the presence of somatic mutations in *APC/CTNNB1* genes is a prognostic factor in cutaneous melanoma, we performed OS analysis on the TCGA SKCM cohort [17]. Fig. 1A shows the CONSORT diagram of the 470 specimens from 470 patients that we analyzed for *APC*/*CTNNB1* somatic mutation status. Unfortunately, we could not classify 36 samples due to missing data (unknown *tumor_tissue_site*, missing *curated_days_to_last_followup*, unknown *curated_pathologic_stage* in samples that were not distant metastases). The CONSORT diagram from Fig. 1A shows the distribution of *APC*/*CTNNB1* somatic mutations according to the tumor tissue site and clinical AJCC at specimen collection.

Table 1 shows the histopathologic characteristics of cutaneous melanoma samples according to somatic *APC*/*CTNNB1* gene mutation status. There were no differences in the Breslow depth of invasion and mitotic rate in *APC/CTNNB1* wild-type compared to mutant tumors; the only exception was the higher incidence of ulceration of the primary *APC/CTNNB1 *wild type melanomas upon the original diagnosis. There was no correlation between *APC*/*CTNNB1* gene somatic mutation status and lymphocyte score, a measure of lymphocyte density and distribution (peritumoral and intratumoral) performed as part of the TCGA SKCM by consensus review among six expert melanoma pathologists [17]. Across the 470 tumor specimen cohort, we did not identify deep deletions in the *APC* gene (< 0.33 copies/mean_cancer). We identified a single specimen with *CTNNB1* gene amplification (> 2 copies; TCGA-D3-A3BZ-06); nevertheless, a handful of specimens exhibited relative copy gains/losses (≥ 50% or ≤ 50% of ploidy). RNA sequencing analysis revealed that both *APC* and *CTNNB1* mutations were significantly expressed. Mutation co-occurrence analysis showed that no somatic mutations in other melanoma-associated genes were significantly correlated with *APC*/*CTNNB1* somatic mutations after controlling for tumor mutation burden and multiple testing comparisons (data not shown).

**Table 1:** Pathologic and clinical data of patients from the Cancer Genome Atlas Project in Cutaneous Melanoma (TCGA-SKCM) according to the *APC/CTNNB1* somatic mutation status. Abbreviations: * *CTNNB1* copies/mean_cancer_ploidy_rounded_to_nearest_integer

	***APC/CTNNB1*** Mutant ***N*** = 55	***APC/CTNNB1*** Wild type ***N*** = 415
Age, at specimen procurement (years, median, range)	64 (37, 90)	61 (19, 90)
Breslow Depth, original diagnosis (mm, median, range)	2.85 (0.25, 15)	3 (0, 75)
Ulceration, original diagnosis (percent of specimens %)	**39**	**55**
Mitotic count rate, original diagnosis (mitoses/mm^2^, median, range)	4 (0, 33)	5 (0, 40)
Lymphocyte Score, procured specimen (1+ thru 6+ median, range)	2+ (0,6)	2+ (0, 6)
*CTNNB1* gene copies* (median, range)	1 (0.5, 1.5)	1 (0.5, 3)
APC gene copies* (median, range)	1 (0.5, 2.5)	1 (0.33, 2.5)

*APC*/*CTNNB1* somatic mutations were not associated with adverse prognosis in patients with stage II SKCM (*n* = 66). The median OS of patients bearing mutant vs. wild-type *APC*/*CTNNB1* melanomas was 25.85 vs. 42.5 months (log-rank hazard ratio [HR] 1.42, 95% confidence intervals [95CI] 0.22–12.57, *p* = 0.69). Out of 286 patients with clinical stage III SKCM at specimen procurement, 36 [12.5%] patients had *APC/CTNNB1* mutations. The median OS of patients with *APC*/*CTNNB1*-mutant melanomas trended to be significantly shorter compared to melanomas without *APC*/*CTNNB1* mutations (29 vs. 36.3 months, log-rank HR 1.57, 95CI 0.92–2.69, *p* = 0.099). Eighty-two patients from the TCGA SKCM cohort were clinically staged as IV (MM) at specimen procurement. Of these, only eight patients (9.8%) had *APC/CTNNB1* mutations. The median OS of patients with *APC*/*CTNNB1*-mutant melanomas was significantly shorter than that of patients bearing wild-type *APC*/*CTNNB1* melanomas (8.15 vs. 22.8 months, log-rank HR 4.2, 95CI 1.38–12.58, *p* = 0.011). Figure 1B shows corresponding Kaplan-Meier curves of patients with mutant vs. wild-type *APC*/*CTNNB1* melanomas according to clinical AJCC stage. **Supplementary File** **1****, Fig. S2,** shows oncoplots corresponding to the 82 tumor tissues from patients with stage IV melanoma. When the data from the 82 patients with stage IV melanoma were fitted into a Cox proportional hazard model that included the *APC*/*CTNNB1* mutation status and the mutation burden as covariates, the *APC*/*CTNNB1* mutation status coefficient remained significant (*p* = 0.0245).

### Patient characteristics bearing melanomas with *APC*/*CTNNB1* mutations (UNC-CH/Vanderbilt/California Pacific medical research institute)

Given that in the TCGA SKCM cohort *APC*/*CTNNB1* mutations are infrequent but have an adverse prognosis only in metastatic cutaneous melanoma, we sought to investigate their theragnostic significance in a much larger and more contemporary MM patient cohort with known *APC*/*CTNNB1* mutations and measurable disease. The combined UNC-CH/Vanderbilt/CPMRI included tumors from 676 patients with any stage melanoma between Oct 2006 and April 2021. Of these, 55 patients’ tumors who either eventually developed or originally presented with MM contained *APC* or *CTNNB1* genetic aberrations (8.1%). Table 2 shows the demographics, clinical, and molecular characteristics of all patients with MM with (*n* = 55) and a subset of patients without (*n* = 169) *APC*/*CTNNB1* genetic aberrations. **Supplementary File** **2** shows individual patient data. There were no significant differences in the demographics and melanoma subtypes between the two MM patient cohorts. The majority of patients were males (approximately 60%), with a median age of 61 years and a diagnosis of cutaneous melanoma (approximately 75%).

**Table 2:** Demographics, clinical, and pathologic characteristics of the UNC-CH/Vanderbilt/California Pacific Medical Research Institute

	APC/CTNNB1 mutant	APC/CTNNB1 wild type
**Characteristics**	**Total (*n*** **= 55, %)**	**Total (*n*** **= 169, %)**
**Sex**		
Male (%)	35 (64)	99 (59)
Female (%)	20 (36)	70 (31)
**Melanoma Type**		
Cutaneous (%)	42 (76)	123 (73)
Acral (%)	5 (9)	15 (9)
Mucosal (%)	1 (2)	14 (8)
Uveal (%)	1 (2)	0 (0)
Unknown Primary (%)	6 (11)	12 (7)
No information available (%)	0	5 (3)
**Age** at MM diagnosis median (range in years)	61 (27–80)	61 (21–99)
**Next Generation DNA sequencing Assay**		
Illumina 26-gene panel	23 (42)	0
FoundationOne CDx	32 (58)	169 (100)
**Development of Brain Metastases**		
**Yes (%)**	**24 (44)**	**66 (39)**
Time to development from MM diagnosis (median, range in months)	1.8 (0,96)	8.6 (0,106.4)
**No (%)**	**31 (56)**	**103 (61)**
**Systemic Treatments-Immunotherapies**		
**Yes (%)**	**48 (87)**	**151 (89)**
Response (%)	27 (56)	63 (42)
Progression (%)	20 (42)	86 (57)
No information (%)	1 (2)	2 (1)
**No (%)**	**7 (13%)**	18 (11)
**Immunotherapy Types**		
Ipilimumab alone (%)	1 (2)	1 (1)
PD1 inhibitor alone (%)	23 (48)	90 (6)
Ipilimumab plus PD1 inhibitors (%)	24 (50)	59 (39)
High dose bolus IL-2 (%)	1 (2)	10 (7)
Other (IFNα2b) (%)	3 (6)	1 (1)
**Systemic Treatments-Non-immunotherapies**		
BRAF inhibitors and/or MEK inhibitors (%)	16 (29)	42 (25)
Other targeted therapies (%)	5 (9)	14 (8)
Chemotherapies (%)	6 (11)	25 (15)
**Genetic aberrations**		
Number of mutations/Mb* (median, range) **APC/CTNNB1 genetic aberrations**	20 (2,372)	13 (0,160)
*CTNNB1* alone (%)	29 (53)	N/A
*APC* alone (%)	25 (45)	N/A
Both *CTNNB1* and *APC*	1 (2)	N/A
**Other mutations**		
***BRAFV600*** **(%)**	**19 (35)**	**47 (28)**
*V600E*	16 (29)	40 (24)
*V600K*	3 (5)	6 (4)
*V600D*	0	1 (1)
***BRAFK601*** **(%)**	**1 (2)**	**2 (1)**
***NRASQ61*** **(%)*1***	**17 (31)**	**36 (21)**

At a median follow-up of 26.1 months from the original diagnosis of MM (range 0.6–156.6 months), 25 patients (45%) with MM and *APC*/*CTNNB1* genetic aberrations had died from MM. At a median follow-up of 28.5 months from the original diagnosis of MM (range 1–210 months), 99 patients (58.6%) with no *APC*/*CTNNB1* genetic aberrations had died from MM. The incidence of brain metastases in patients with MM and *APC*/*CTNNB1* genetic aberrations was slightly higher than that in patients without (44% vs. 39%). Furthermore, 66% of patients with *APC*/*CTNNB1* genetic aberrations developed brain metastases within six months from the original diagnosis of MM compared to 45% of patients without; however, the time-to-development brain metastases was not significantly different between the two groups (data not shown).

**Fig. 2:**
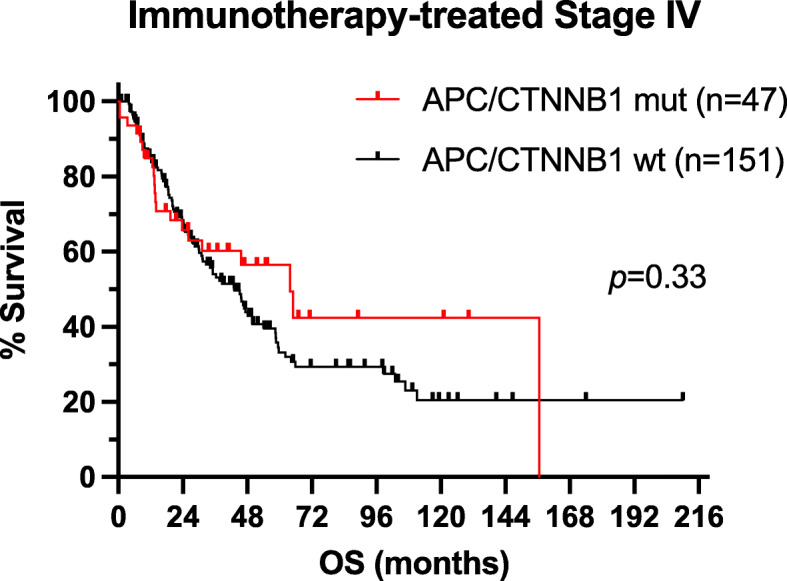
Overall survival (OS) analysis of patients with metastatic melanoma who have received immunotherapies at some point during the natural history of their disease according to the *APC*/*CTNNB1* genetic aberration status (combined UNC-CH/Vanderbilt/CPMRI cohort). Please note that one subject from the *APC/CTNNB1*-mutant group was lost to follow-up

More than 85% of patients in both cohorts received immunotherapies, particularly PD1/PD-L1 and/or CTLA4 inhibitors. Although the percentage of patients who received immunotherapies was similar in both cohorts, more patients in the *APC*/*CTNNB1*-mutant cohort received ipilimumab plus PD1 inhibitors (50% vs. 39%). The incidence of patients receiving BRAF and/or MEK inhibitors or other targeted therapies was similar in both cohorts. The overall antitumor response to immunotherapies in patients with MM and *APC*/*CTNNB1* genetic aberrations was higher than that in patients without (56% vs. 42%). However, the OS of patients with MM and *APC*/*CTNNB1* genetic aberrations who received immunotherapies was not significantly different from that of patients without (median OS 26.1 months vs. 29.9 months, respectively, log-rank *p* = 0.33). Fig. 2 shows OS analysis (Kaplan-Meier method) for the two MM patient cohorts that have received immunotherapies, according to the *APC*/*CTNNB1* genetic aberration status.

**Fig. 3:**
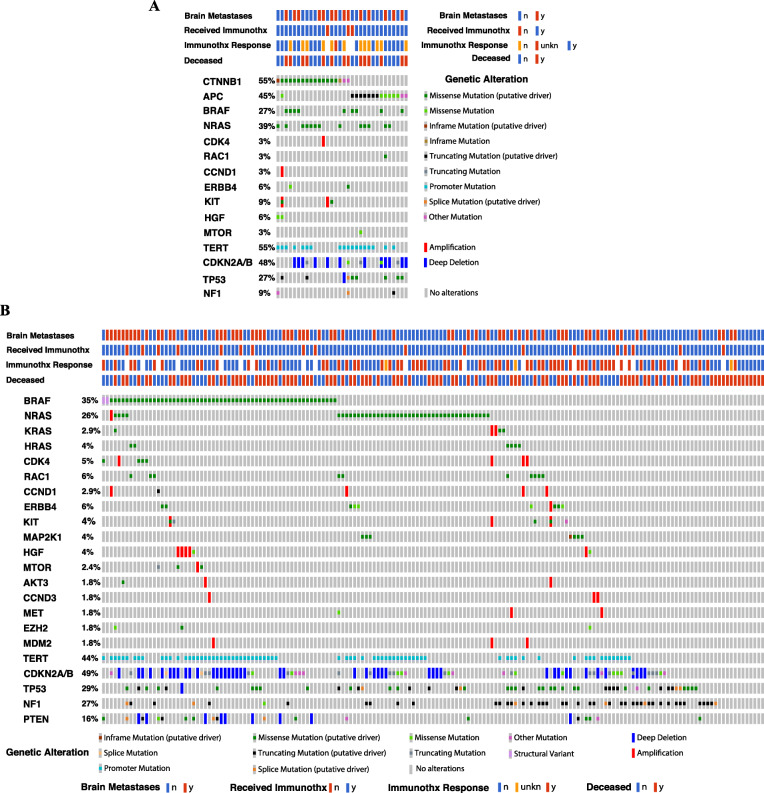
Clinical data and genetic aberrations in *APC*, *CTNNB1*, and other melanoma-associated genes in melanoma tissues that were sequenced with the Foundation One CDx assay from the combined UNC-CH/Vanderbilt/CPMRI patient cohort. Patient subsets with *APC*/*CTNNB1* genetic aberrations (*n* = 32, panel A) and without *APC*/*CTNNB1* mutations (*n* = 169, panel B) are shown. Abbreviations: Immunothtx, immunotherapies; n, no; y, yes; unkn, unknown

58% of patients’ tumors with and all (100%) patients’ tumors without *APC*/*CTNNB1* genetic aberrations were sequenced with the FoundationOne CDx assay. Fig. 3 shows OncoPrint plots for the *APC* and *CTNNB1* genes as well as other oncogenes and tumor suppressor genes that frequently undergo genetic aberrations in MM [17]. **Supplementary File** **2** shows all reported genetic aberrations in individual patients. Except for one tumor specimen (subject 43), *APC* and *CTNNB1* genetic aberrations were mutually exclusive. Two tumor specimens harbored two different mutations for each of the *APC* and *CTNNB1* genes (subjects 6 and 37, respectively). 35% of tumor specimens with *APC*/*CTNNB1* genetic aberrations did not harbor either *BRAFV600/K601* or *NRASQ61* mutations compared with 50% of tumor specimens without. We then directly compared genetic aberrations in other genes between the two patient cohorts who underwent targeted panel sequencing using the FoundationOne CDx assay only (32 tumors from the *APC*/*CTNNB1-*mutant group and all 169 tumors from the *APC*/*CTNNB1*-wild type group). We found that the incidence of genetic aberrations in the *CDKN2A/B* locus genes, *CCND1*, *CDK4*, *ERBB4*, *HGF*, *MTOR*, and *TP53* genes was similar in both groups. However, the frequency of genetic aberrations in the *NF1*, *RAC1*, and *PTEN* genes was less in the *APC*/*CTNNB1*-mutant specimens. Finally, patients with *APC*/*CTNNB1* genetic aberrations had a slightly higher incidence of *TERT* promoter mutations (55% vs. 44%).

### Total β-catenin is abundantly expressed in established melanoma brain metastases, but activated β-catenin is not

Our findings regarding the slightly higher incidence and earlier (i.e., within six months) development of MBMs in patients with *APC*/*CTNNB1* genetic aberrations than patients without led us to hypothesize that *APC*/*CTNNB1* genetic aberrations may play a role in the early development of MBM through β-catenin activation. We, therefore, investigated the β-catenin protein expression in tissue sections from patients who underwent craniotomy for MBMs. In addition, assuming that nuclear localization of β-catenin within melanoma cells is a surrogate marker for β-catenin pathway activation, we asked whether activation is more frequent in MBMs devoid of TILs than patients with a high density TILs.

**Fig. 4:**
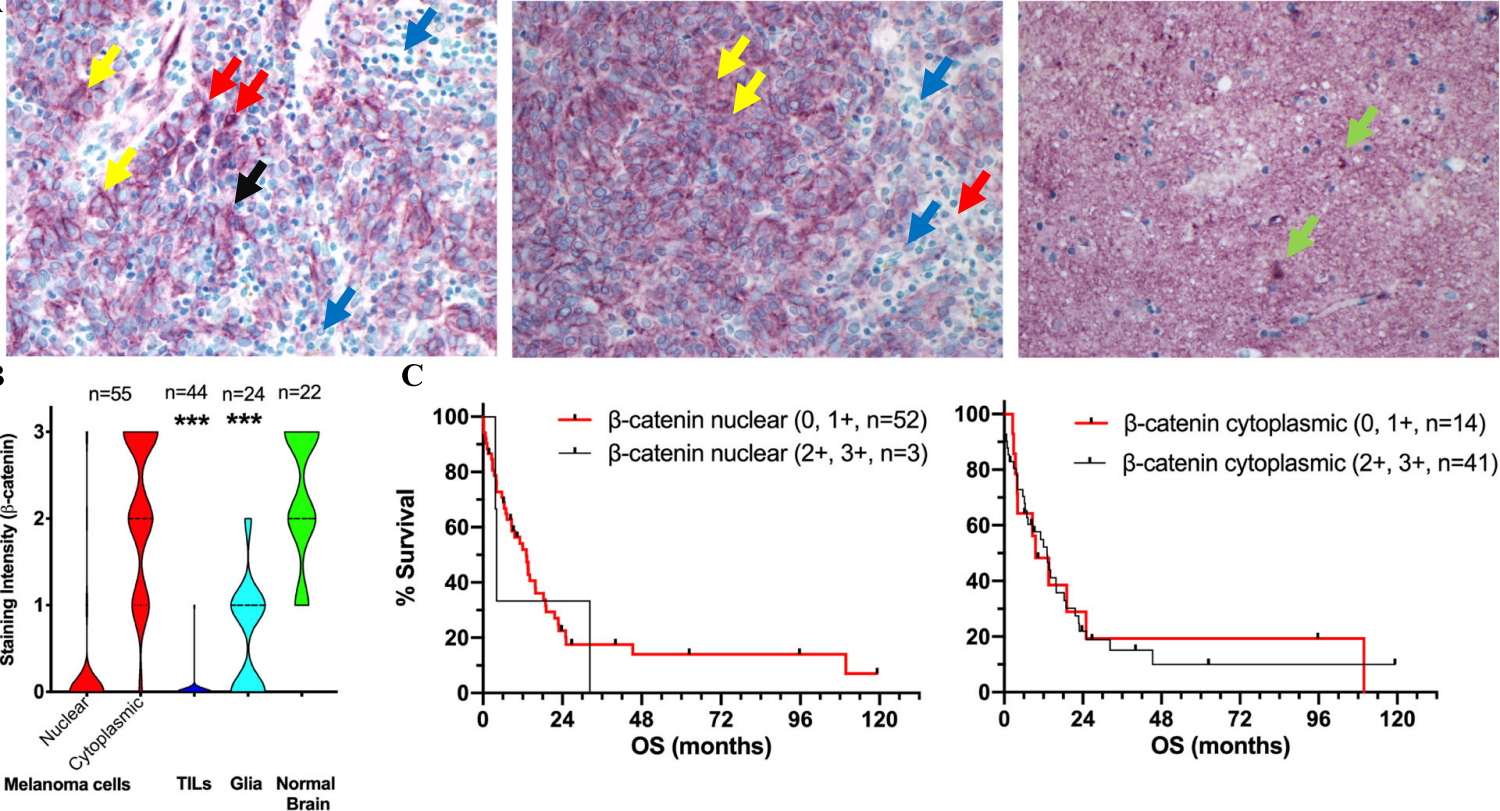
Expression of β-catenin in melanoma brain metastases. (**A**) Digital images (40X magnification) corresponding to representative tissue sections obtained from craniotomy specimens that were immunohistochemically stained with an antibody against β-catenin (ImmPACT VIP, purple; methyl green, cyan). Examples of melanoma cells expressing β-catenin in the nucleus (3+, upper left, red arrows) and, cytoplasm (3+, upper left, yellow arrows; 2+, center left, yellow arrows). Tumor-infiltrating lymphocytes (TILs, upper left and center left, blue arrows) do not express β-catenin whereas neurons (upper right, green arrows) have strong β-catenin expression. (**B**) Expression of β-catenin in different cell compartments (melanoma cells, TILs, glia cells, and adjacent normal brain tissue). Wilcoxon test was performed to compare β-catenin expression between melanoma cells and other brain compartments. Numbers indicate number of observations. (**C**) Overall survival analysis (Kaplan-Meier method) of patients who underwent craniotomy according to β-catenin status (high, low) and localization (nuclear, cytoplasmic); Abbreviations: OS, overall survival; TILs, tumor-infiltrating lymphocytes; *p*-value < 0.001***

The craniotomy cohort included 55 patients (37 males, 67%). The median age at the time of craniotomy was 55 years (range 31–87 years). Only 7 out of 55 (13%) patients received targeted therapies or immunotherapies following craniotomy. Of note, the *APC*/*CTNNB1* somatic mutation status for this patient cohort was unknown. Nearly all (96%) tumors expressed cytoplasmic β-catenin within melanoma cells; however, 5% (3/55 patients) also expressed strong (2+, 3+) nuclear β-catenin (Fig. 4A). Expression of cytoplasmic β-catenin was significantly higher in melanoma cells than in adjacent TILs and reactive glia, but was not significantly different from the expression in adjacent non-neoplastic brain cells (Fig. 4B). Neither cytoplasmic nor nuclear β-catenin expression in melanoma cells significantly correlated with TIL density (data not shown). At a median follow-up of 9.6 months (range 0.1–119.3 months), 80% of patients had expired from MM. The OS of patients with high (2+, 3+) protein expression of β-catenin (nuclear, cytoplasmic) was not significantly different compared to that of patients with lower (0, 1+) β-catenin (Fig. 4C).

## Discussion

This study investigated the clinical (i.e., theragnostic) significance of *APC*/*CTNNB1* genetic aberrations across two clinicopathologically annotated melanoma patient cohorts. The first cohort (TCGA SKCM) included patients with stage II-IV melanoma, was not enriched for *APC*/*CTNNB1* mutations (approximately 11.5%, prospective analysis), and historically preceded FDA approval of PD1 inhibitors. The second cohort only included patients with stage IV melanoma who were predominantly (> 85%) treated with PD1 inhibitors across three US melanoma institutions and was enriched for patients with *APC*/*CTNNB1* mutations (25%, retrospective chart review analysis). The study’s significant findings are that *APC*/*CTNNB1* somatic mutations have adverse prognosis in later stages of melanoma. Not surprising for genetic aberrations in genes that have an adverse prognosis in stage IV melanoma [14–16], we also found that patients with *APC*/*CTNNB1* genetic aberrations and stage IV melanoma have slightly higher incidence and earlier onset (i.e., within six months of diagnosis of MM) of MBMs compared to patients without *APC*/*CTNNB1* genetic aberrations. Nevertheless, the presence of *APC*/*CTNNB1* genetic aberrations in stage IV melanoma does not diminish the clinical benefit from immunotherapies. Our study’s strengths are our complementary (i.e., genetic and immunohistochemical) investigations of the β-catenin pathway in melanoma samples across independent patient cohorts. Nevertheless, each patient cohort had its limitations in data interpretation and generalizability of findings, in part related to the low incidence of *APC*/*CTNNB1* genetic aberrations in melanoma. Previous patient cohorts have focused on direct analysis of β-catenin signaling concerning prognosis and histopathology [11, 28].

Analysis of the prognostic significance of somatic *APC*/*CTNNB1* mutations from the entire TCGA SKCM cohort provided a direct comparison between melanoma patients with or without *APC*/*CTNNB1* somatic mutations. We found that *APC* and *CTNNB1* somatic mutations do not significantly coexist with somatic mutations in other melanoma-associated genes after controlling for somatic tumor mutation burden. Under the critical assumption that *APC* and *CTNNB1* somatic mutations are not ‘passenger’ but play an essential role throughout the natural history of melanoma, irrespective of the tumor tissue site and the curated pathologic stage, we assessed the role of *APC*/*CTNNB1* somatic mutations in each clinical AJCC stage. Our OS analysis suggests that *APC*/*CTNNB1* somatic mutations may have some role in regional metastatic and, even more so, in distant metastatic disease. Given the low frequency of somatic *APC*/*CTNNB1* mutations in melanoma (approximately 10–12% across all stages), the comparator arms were largely unbalanced, which may be the reason why *APC*/*CTNNB1* somatic mutations were associated with worse prognosis in stage IV, and only trended towards significance in stage III melanoma. To understand the mechanism underlying the adverse prognosis of somatic *APC*/*CTNNB1* mutations in MM, we sought to investigate the association between the density of TILs and *APC*/*CTNNB1* somatic mutation status given previous reports between immune exclusion and activation of the Wnt/β catenin pathway across various cancers [9]. To our surprise, we did not find any correlation between *APC*/*CTNNB1* somatic mutation and lymphocyte score in the TCGA SKCM cohort, a consensus and composite measurement of the density of peritumoral and intratumoral TILs, based on the hematoxylin and eosin analysis of representative tissue sections from the TCGA SKCM melanoma specimens [17]. We, therefore, must assume that *APC*/*CTNNB1* somatic mutations may have differential effects in cancer cells other than by merely activating β-catenin [29].

The retrospectively compiled 3-institution MM cohort is, to our knowledge, the largest ever reported, clinicopathologically annotated database comprised of patients who have been predominantly treated with PD1 inhibitors, and their melanoma tumors have undergone targeted panel sequencing. Identification of 55 patients with stage IV melanoma with *APC*/*CTNNB1* genetic aberrations allowed us to understand their role in predicting response to immunotherapies more robustly than the eight, stage IV patients with *APC*/*CTNNB1* somatic mutations from the TCGA SKCM cohort. This analysis, however, was challenged with additional limitations related to differences in mutation calling and exon coverage between the two different targeted panel sequencing methods, TruSight Tumor 26-gene Illumina and FoundationOne CDx assay. A further limitation with the second cohort is an inherent inability to differentiate out germline from somatic mutations when the standard of care targeted panel sequencing data is considered. Based on a published catalog of statistically significant hotspot mutations in cancer [23], however, nearly all missense *CTNNB1* mutations in this cohort were in hotspots. In contrast, none of the missense *APC* mutations were hotspot mutations. Also, nearly all missense *APC* gene mutations from the FoundationOne CDx cohort in which sequencing covered all *APC* gene exons either fell within exon 15 or within regions corresponding to other relevant functional domains (e.g., P2622 and PS2631 codons within the EB1 binding domain). For example, the recurrent I1307K mutation that we saw in three patients is a hotspot for increased colorectal cancer risk (and presumably germline because we did not see it in the TCGA SKCM cohort) [30]. Noteworthy was also the observation from the 3-institution cohort that genetic aberrations of specific melanoma-associated genes were less frequent in *APC*/*CTNNB1*-mutant stage IV melanomas than the incidence of these mutations without (e.g., *NF1*, *RAC1*, and *PTEN*). Although selection bias can account for this interesting finding in this retrospective chart review analysis, the incidence of genetic aberrations in other melanoma-associated genes was not different (e.g., *CDKN2A/B* locus). We, therefore, conclude that the overwhelming majority of *APC*/*CTNNB1* genetic aberrations are functionally significant and may contribute to the activation of the β-catenin signaling pathway, which may be per se essential for melanoma progression even in the absence of activation of other signaling pathways regulated by tumor suppressor genes, such as *NF1* and *PTEN*.

In contrast with preclinical data suggesting that activation of the β-catenin pathway in melanoma cells associates with resistance to anti-PD-L1/anti-CTLA-4 antibody therapy [11], we found that more than 50% of patients with *APC*/*CTNNB1* genetic aberrations responded to immunotherapies. The percentage of patients without *APC*/*CTNNB1* genetic aberrations who responded to immunotherapies was admittedly less; however, more patients with *APC*/*CTNNB1* genetic aberrations happened to receive combined PD1 and CTLA4 inhibitors in this retrospective chart review analysis. Furthermore, the OS of patients with *APC*/*CTNNB1* genetic aberrations was not significantly different from the OS of patients without. We believe the following reasons may explain the discrepancy between our findings regarding clinical benefit from immunotherapies in patients with *APC*/*CTNNB1*-mutant melanoma and previous preclinical or translational observations [11, 31]. First, the melanoma syngeneic mouse model treated with PD-L1 inhibitors had a specific genetic background (Braf^V600E^/Pten^−/−^). Second, in the mouse model, β-catenin stabilization involved the targeted excision of the *entire CTNNB1* exon 3 [32]. The biologic implications of this genetic manipulation (i.e., β-catenin stabilization) may differ from the effect of *CTNNB1* mutations, causing a change in a single amino acid within exon 3. Third, for *CTNNB1* exon 3 mutations, previous studies have shown that different hotspot *CTNNB1* mutations result in different levels of β-catenin activation and differential association of β-catenin with other multiprotein complexes (e.g., transcription, destruction, or adhesion complexes) [29]. Therefore, it remains to be seen whether specific *APC*/*CTNNB1* genetic aberrations variably influence β-catenin stability and ultimate function within the cells in a fashion possibly beyond mere β-catenin activation. The findings may challenge ‘linear’ thinking that *APC*/*CTNNB1* genetic aberrations consistently lead to β-catenin pathway activation.

Perhaps the most intriguing finding was the slightly more frequent and earlier development of MBMs in patients with *APC*/*CTNNB1* genetic aberrations who developed MM in the combined UNC-CH/Vanderbilt/CPMRI cohort compared to those patients without *APC*/*CTNNB1* genetic aberrations. Earlier and slightly more frequent progression to M1d disease, the prognostically worst M1 substage based on the recent revision of the AJCC staging system [21], may in part account for the worse OS of patients with *APC*/*CTNNB1* genetic aberrations and stage IV melanoma that we saw in the TCGA SKCM dataset. The 3-institution cohort includes Foundation Onc CDx data from 201 patients with stage IV melanoma and is the largest ever reported patient database that has recorded MBMs with mature follow-up (median follow-up longer than two years). In this cohort, the incidence of MBMs was 40% (80 out of 201). Patients with MBMs as opposed to patients without had more frequent genetic aberrations in *BRAFV600*/*K601* (40% vs. 21%), *CDKN2A*/*B* locus (55% vs. 45%), *PTEN* (17.5% vs. 9%), *KIT* (6% vs. 3%), *CDK4* (6% vs. 3%), *SETD2* (6% vs. 3%), and *IDH1* genes (5% vs. 2%, **Supplementary File**). Nevertheless, analysis of co-occurring mutations in the 14 patients with *APC*/*CTNNB1* mutations and ΜΒΜs from the 3-institution cohort suggests that four of them (29%) did not have mutations in *BRAFV600*, *CDKN2A/B* locus*, PTEN*, *RAC1*, and *KIT* genes, suggesting that *APC*/*CTNNB1* genetic aberrations per se may have an independent, yet weak, role in the development of MBMs. Nevertheless, activated Wnt/β-catenin signaling in cancer cells is essential for epithelial-mesenchymal transition, migration, and invasion [33]. Frequent genetic aberrations in the *APC* gene occur in brain metastases from various solid tumors [34], whereas infrequent (< 10%) genetic deletion or hypermethylation of the *APC* gene occurs in MBMs [35–37]. We conclude that *APC*/*CTNNB1* genetic aberrations may cooperate with other essential mutated genes to foster brain metastasis development but may less frequently have such an independent effect.

In contrast with the potential role of *APC*/*CTNNB1* mutations in established brain metastases from other solid cancers [38], our results show rare (5%) activation of β-catenin. Furthermore, neither nuclear (i.e., activated) nor cytoplasmic melanoma-intrinsic β-catenin abundance had any prognostic significance in a large cohort of patients who underwent craniotomy for MBMs. It is important to emphasize that most patients in the craniotomy cohort did not receive any immunotherapies following craniotomy. In agreement with a previous report [39], we did not find any association between β-catenin expression and immune infiltration. Of note, in this patient cohort, the incidence of melanoma-intrinsic activated β-catenin signaling was significantly lower than previously described in MM [40]. Although differences in sensitivity in detecting nuclear localization of β-catenin may account for this discrepancy, the β-catenin signaling pathway may not play an essential role once MBMs have been established. We thus conclude that although *APC*/*CTNNB1* mutations may contribute to the development of parenchymal brain metastases, melanoma-intrinsic β-catenin signaling plays a less significant role.

We conclude that *APC*/*CTNNB1* genetic aberrations in patients with established MM are associated with shorter OS than patients without *APC*/*CTNNB1* genetic aberrations. *APC*/*CTNNB1* genetic aberrations are not enriched in non-inflamed melanomas. *APC*/*CTNNB1* genetic aberrations as a whole are not poor predictors of response to PD1 inhibitor-based treatments. Patients with MM and *APC*/*CTNNB1* genetic aberrations who have received PD1 inhibitors at some point during their disease’s natural history have a similar OS with that of patients without *APC*/*CTNNB1* genetic aberrations. The phenomenon of a genetic aberration that may be associated with a worse prognosis if no effective systemic treatments are administered is reminiscent of the clinical significance of *BRAFV600* mutations in MM; although *BRAFV600* mutations are associated with worse outcomes, treatment with a BRAF inhibitor may improve outcome to the degree that is similar to that of patients without *BRAFV600* mutation [16]. These findings do not per se contradict previous preclinical reports [11], because different *APC*/*CTNNB1* genetic aberrations may variably regulate β-catenin association with various competing multiprotein complexes within melanoma cells, and therefore various tumor progression events (**Supplementary File, Fig. S1**) [29].

## Supplementary Information

**Additional file 1: Supplementary Data.**

**Additional file 2.** Individual Patient Data for the UNC-CH/Vanderbilt/California Pacific Medical Research Institute. Abbreviations: F, female; M, male; MM, metastatic melanoma; f/u, follow-up; immunotx, immunotherapy; n/a, non applicable; n/a(CNA), copy number alteration not tested; †, ‡, § next generation sequencing of corresponding genes from the Foundation One CDx assay was not tested (Illumina 26-gene panel).

## Data Availability

All data relevant to the study are included in the article or uploaded as supplementary information.

## Abbreviations

95CI: 95% confidence intervals
APC: adenomatous polyposis coli
AJCC: American Joint Committee on Cancer
CPMRI: the California Pacific Medical Research Institute
CTNNB1: catenin β1
HR: hazard ratio
IHC: immunohistochemistry
IRB: institutional review board
MBMs: melanoma brain metastases
MM: metastatic melanoma (stage IV)
OS: overall survival
RT: room temperature
SKCM: skin cutaneous melanoma
TCGA: The Cancer Genome Atlas Project
TILs: tumor-infiltrating lymphocytes
UNC-CH: The University of North Carolina at Chapel Hill
Wnt: wingless-type MMTV integration site family
