# Supplementary material for: The clinical significance of adenomatous polyposis coli (APC) and catenin Beta 1 (CTNNB1) genetic aberrations in patients with melanoma
Source: BMC Cancer. 2022 Jan 5;22:38. doi: 10.1186/s12885-021-08908-z (PMC8734243; doi:10.1186/s12885-021-08908-z)
Supplement: Supplementary file 1 — Additional file 1: Supplementary Data. [file 12885_2021_8908_MOESM1_ESM.docx]

# **Supplementary Data**

## **Supplementary Figures**

### **Figure S1. Physiologic states of WNT/β-catenin pathway in melanoma and nearby stromal cells.**

**(A)** Glycogen synthase kinase 3β (GSK3β) in its unphosphorylated/constitutively active state forms a complex with the adenomatous polyposis coli (APC) tumor suppressor protein, casein kinase 1α (CK1α), and the axin protein and phosphorylates specific serine (33, 37) and threonine (41) residues in the N-terminal region of β-catenin. Phosphorylated β-catenin is rapidly degraded by the ubiquitin-proteasome (ub) pathway. Binding of the Wnt protein to the Frizzled (fzl) transmembrane receptor activates the dishevelled protein (Dvl), which in turn, phosphorylates/inhibits GSK3β activity and prevents phosphorylation/degradation of β-catenin. Accumulation of β-catenin complexes with members of the T-cell factor or lymphoid enhancer +factor and activates expression of Tcf/LEF-regulated target genes. (B) The β-catenin pathway may be active in melanoma cells and result in increased cell proliferation, migration and decreased infiltration of tumors by immune cells. In addiition, melanoma cells may produce and secrete Wnt5a which actiivates β-catenin signaling in nearby dendritic cells and induces immune tolerance by upregulating components of the hynurenine metabolic pathway. Abbreviations: ATF3, activating transcription factor 3; CCL4, C-C motif chemokine ligand 4; IDO, indoleamine 2,3-diooxygenase 1; Bin1, bridging integrator; Wnt, wnt family member ligands; Cox2, cytochrome C oxidase II; PTGS2, prostaglandin-endoperoxide synthase 2; Treg, T regulatory cells.

**(A)**

**(B)**


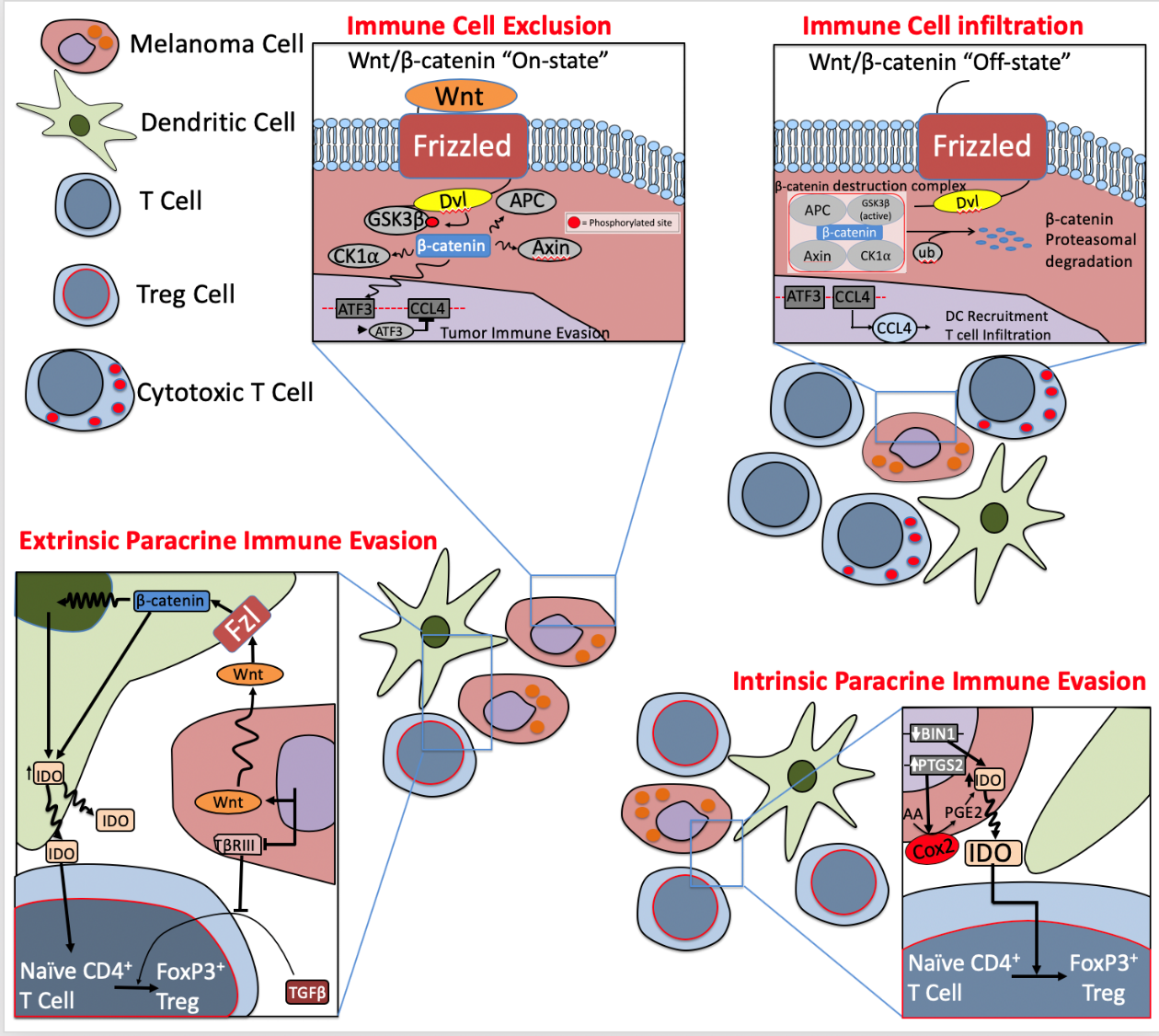


**Figure S2. Somatic mutations in clinical stage IV TCGA SKCM (n=82) with and without *APC* and *CTNNB1* mutations.** Abbreviations: LOF; loss of function; CN, copy number.
